# Supplementary material for: Soil Organic Matter Molecular Composition Shifts Driven by Forest Regrowth or Pasture after Slash-and-Burn of Amazon Forest
Source: Int J Environ Res Public Health. 2023 Feb 16;20(4):3485. doi: 10.3390/ijerph20043485 (PMC9962374; doi:10.3390/ijerph20043485)
Supplement: Supplementary file 1 [file ijerph-20-03485-s001.zip › ijerph-2177407-supplementary.pdf]

Supplementary Materials

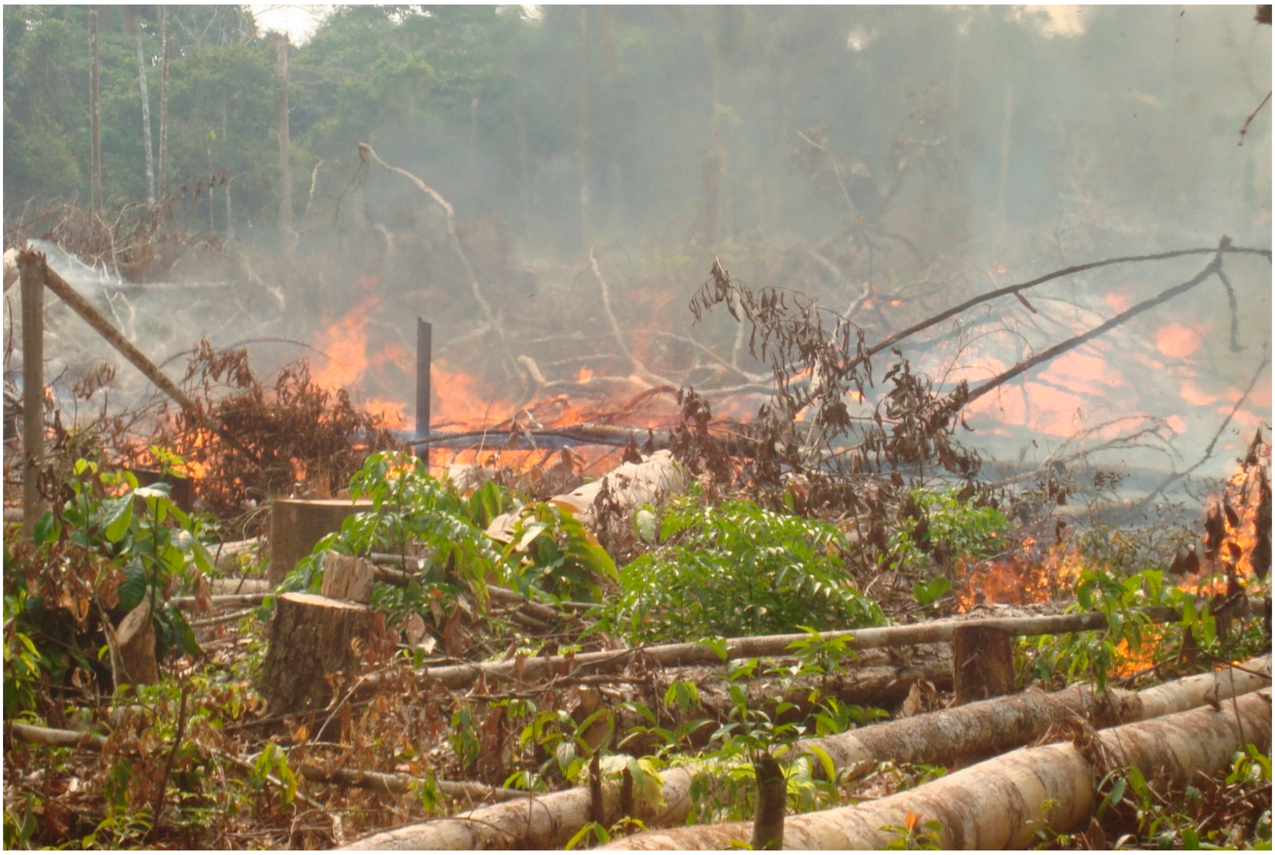

Figure S1. Overview of native Amazon Forest experimental slash-and-burn.

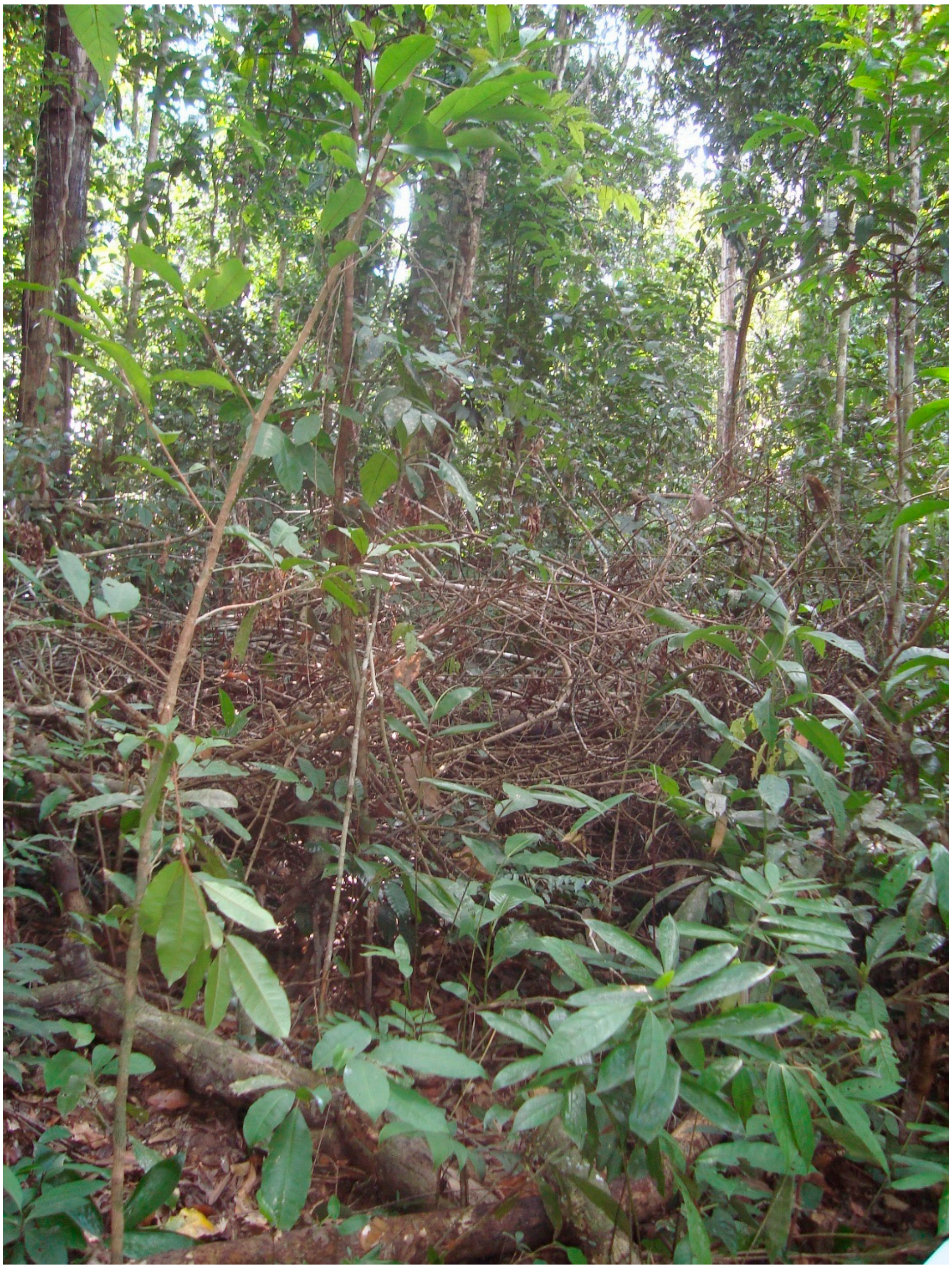

Figure S2. View of the native Amazon Forest study site.

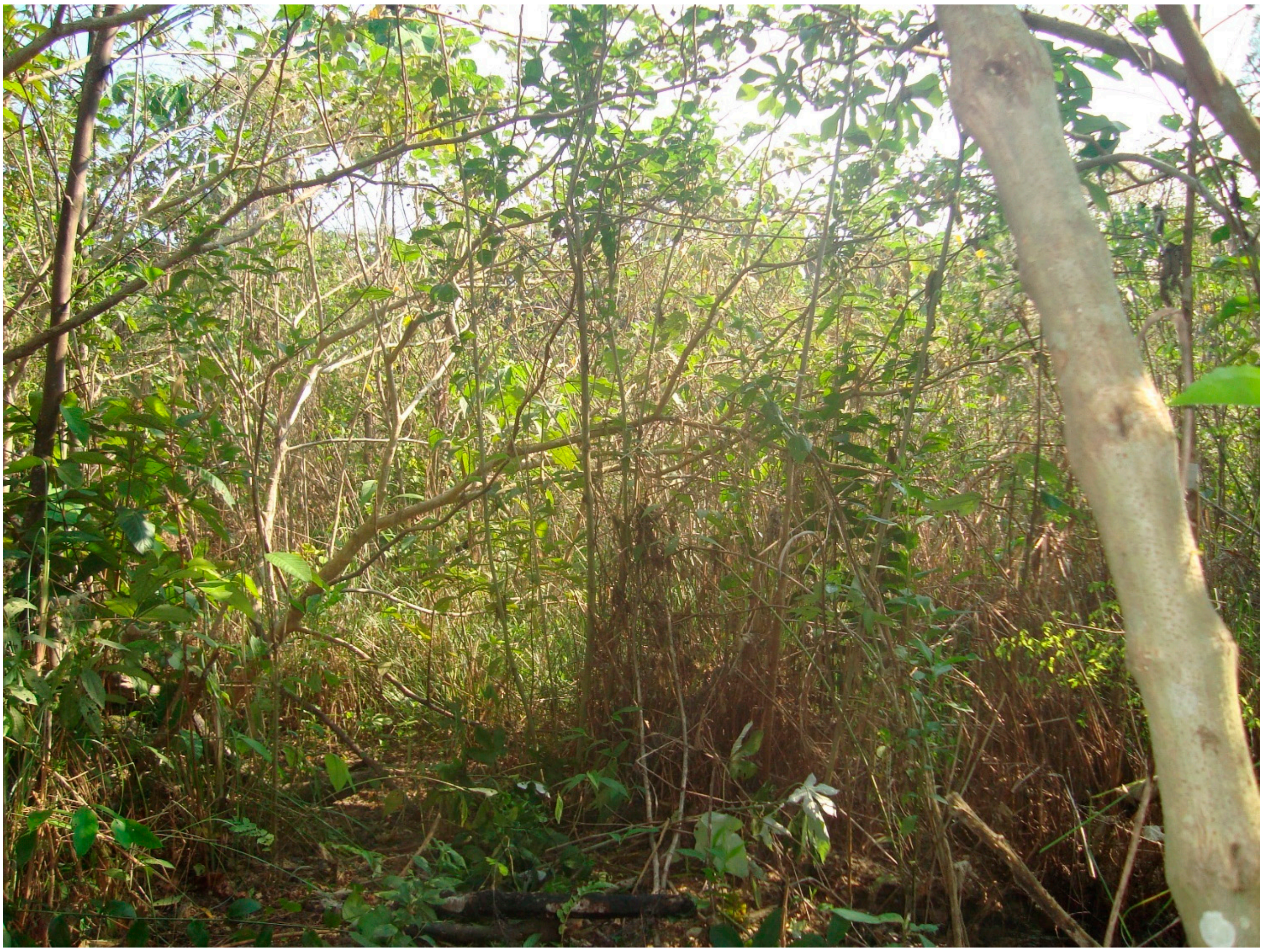

Figure S3. View of the burned Amazon Forest study site 20 months after fire and regrowth of forest.

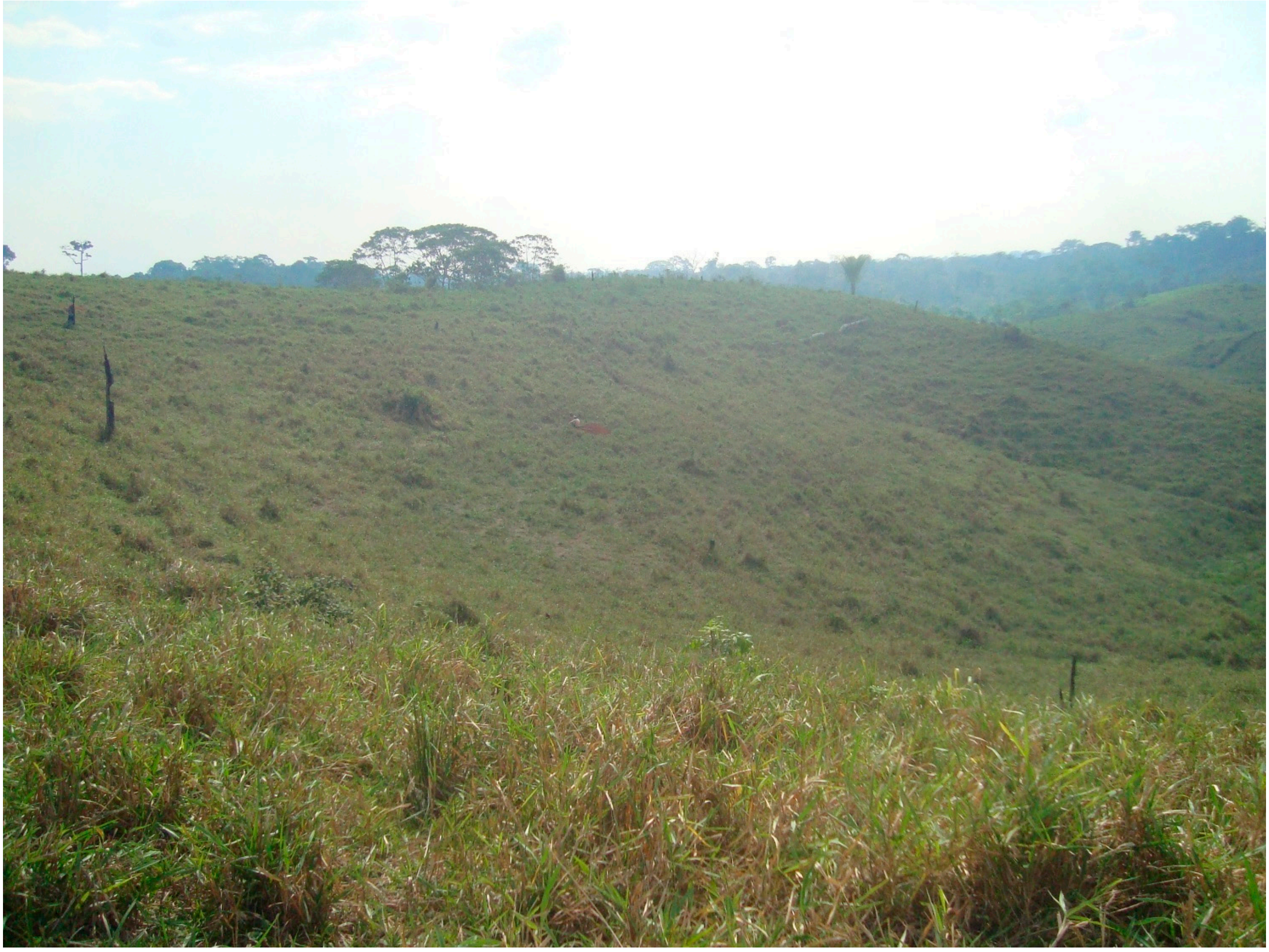

Figure S4. View of the Brachiaria pasture study site.

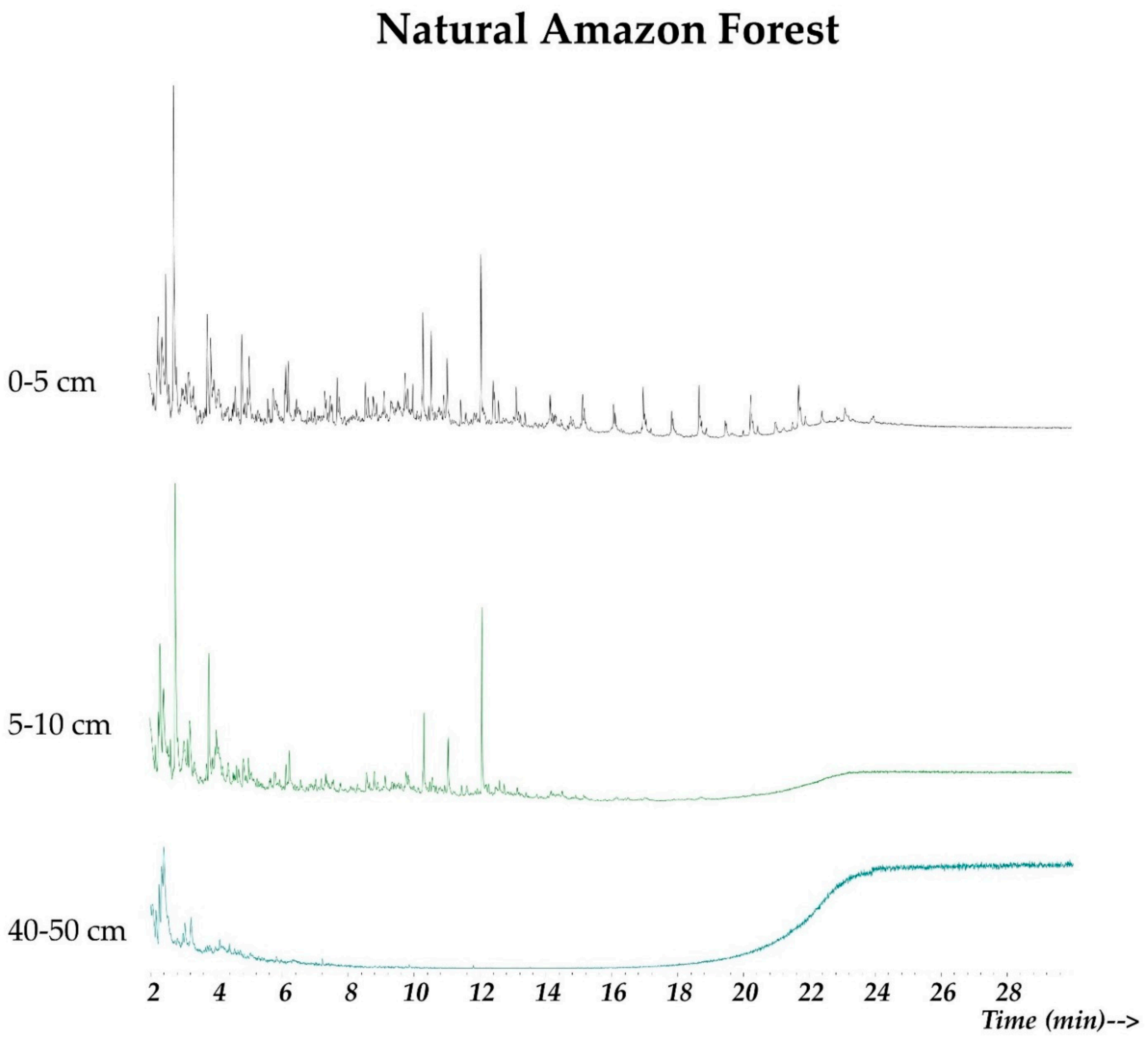

Figure S5. Py-GC/MS total ion chromatogram of native unburned Amazon Forest at 0-5, 5-10 and 40-50 cm soil depth.

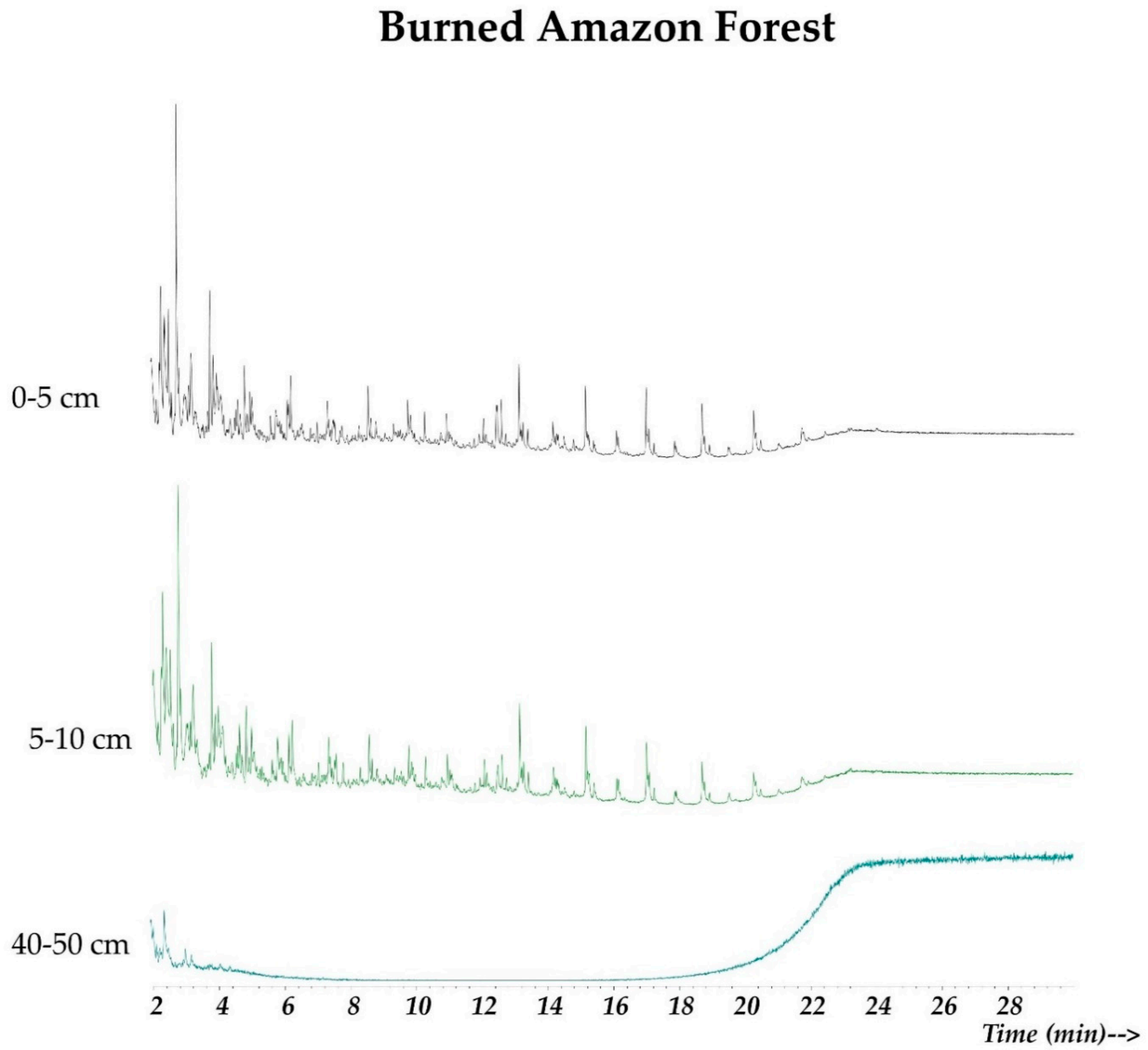

Figure S6. Py-GC/MS total ion chromatogram of burned Amazon Forest at 0-5, 5-10 and 40-50 cm soil depth.

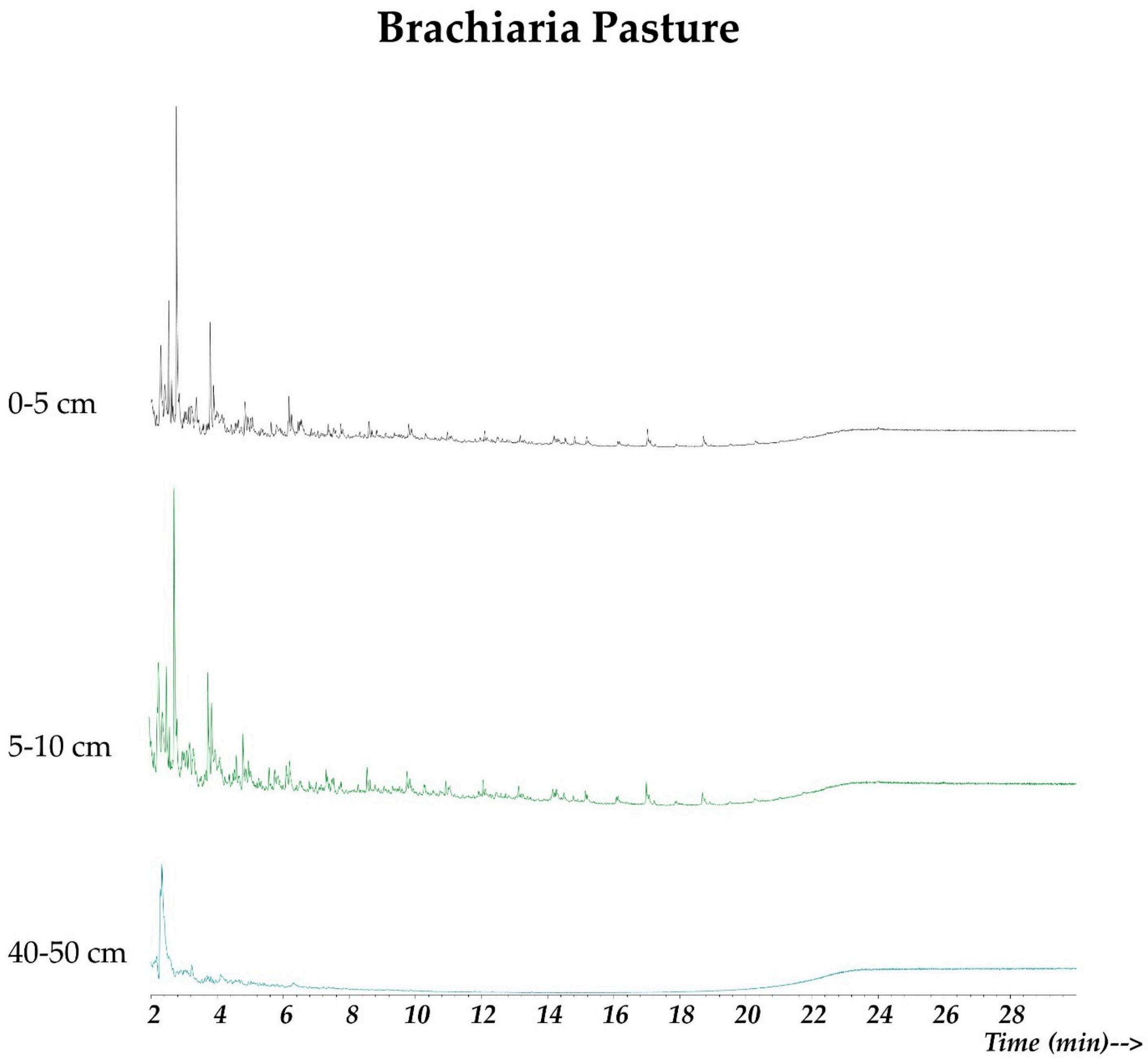

Figure S7. Py-GC/MS total ion chromatogram of Brachiaria pasture at 0-5, 5-10 and 40-50 cm soil depth.

Table S1. List of compounds identified by analytical pyrolysis (Py-GC/MS) in the burned Amazon Forest (BAF), native Amazon Forest (NAF), and Brachiaria (BRA) soil samples at 0-5 cm soil depth.

Family	Rt* (min)	Name of compound	NAF	BRA	BAF
Aromatic	2.0087	Benzene	+++	+++	+++
	2.403	Benzene, methyl-	+++	+++	+++
	3.2089	Styrene	+++	+++	+++
	3.7232	Benzene, propyl-	-	-	+
	3.7861	Benzaldehyde	-	-	+++
	3.8833	Phenol	++	+++	+++
	4.1175	Benzene, 2-propenyl-	-	-	+++
	4.4033	Benzene, 1-ethenyl-4-methyl-	-	-	++
	4.5749	Benzeneacetaldehyde	+	+	+
	4.6376	Benzene, 1-propynyl-	-	+	+
	4.7062	Benzene, butyl	-	-	++
	4.8263	Phenol, 4-methyl-	++	-	-
	4.8319	Acetophenone	-	++	-
	4.8377	Ethanone, 1-phenyl-	-	-	++
	4.9177	3,5-Dihydroxytoluene	-	++	-
	4.9291	1-Phenyl-1-butene	-	-	+
	5.6665	Phenol, 2-(2-methyl-2-propenyl)	+	-	-
	6.0321	Ethanone, 1-(2-methylphenyl)	-	+	-
	6.5523	Benzanamine, 3-ethoxy	-	-	+
	6.6209	1-Phenyl-1-pentene	-	-	+
	6.9409	Benzene, (3-methylcyclopentyl)	-	+	-
	6.941	Benzene, cyclohexyl	+	-	-
	7.0439	Benzene, hexyl	+	-	+
	7.141	2-Phenyl-4-penten-2-ol	-	-	+
	8.307	Benzene, heptyl-	+	+	+
	10.7988	Benzene, nonyl-	-	-	+
	11.9589	Benzene, decyl-	-	+	+
PAH	6.2437	Azulene	++	++	++
	7.7926	Naphthalene, 2-methyl-	++	++	++
	8.5927	Biphenyl	-	-	++
	8.9242	Naphthalene, 1,2-dihydro-1,4,6-trimethyl-	+	-	-
	8.827	1,1-Biphenyl-4-methyl	-	-	+
	9.1528	Naphthalene, 1,6-dimethyl-	+	-	-
	9.3586	6-N-butyl-1,2,3,4-tetrahydronaphthalene	++	-	-
	10.1815	1,1,6,8-Tetramethyl-1,2-dihydro-naphthalene	-	-	+
	10.3072	1,1,4,5,6-Pentamethyl-2,3-dihydro-1H-indene	-	-	+
	12.0962	Naphthalene, 1,6-dimethyl-4-(1-methylethyl)-	++	+	-
	12.4847	2-Methoxy-1,4-dimethyl-naphthyl-naphthalene	-	+	+
	12.5076	Phenanthrene, 1,2,3,4,5,6,7,8-octahydro	-	-	+
	12.7705	1,4,5,8-Tetramethylnaphthalene	-	-	+
	13.4336	Naphthalene, 6-methoxy-2-(1-buten-3-yl)	+	-	-
Lignin	5.0549	Phenol, 2-methoxy	+	++	++
	7.7297	2-Methoxy-4-vinylphenol	+	+	+
	8.8212	Vanillin	-	+	-
	8.8213	Benzaldehyde, 4-hydroxy-3-methoxy-	+	-	-
	9.8672	Ethanone, 1-(4-hydroxy-3-methoxyphenyl)	+	-	-
Lipid	2.5287	1,4-Dioxadiene	-	-	+++
	3.9862	1-Decene	++	++	+++

4.4147	Bicyclo[3.2.0]heptan-2-one	-	++	-
4.489	6-Octen-2-one	-	+	-
5.0035	1-Undecene	+	+	++
6.1408	2-Dodecene	-	-	+
6.1637	1-Hydroxy-3,6-dioxabicyclo[3.2.1]octan-2-one	+	+++	-
7.0381	Bicyclo[6.4.0]dodeca-9,11-diene	-	+	-
7.3525	1-Tridecene	+	++	++
7.3868	Cyclohexane, 2-butyl-1,1,3-trimethyl	+	-	-
7.4438	Tridecane	-	+	+
8.587	1-Tetradecene	+	+	-
8.6727	Tetradecane	+	+	+
9.0955	β-Damascenone	-	+	-
9.3642	1-Pentadecene	+	+	++
9.4328	Pentadecane	-	-	+
9.6043	γ-Muurolene	+	-	-
9.8728	Pentadecane	-	+	++
10.0273	Isoledene	+	-	-
10.3359	*trans*-Calamenene	++	-	-
10.5874	α-Calacorene	++	-	-
10.9646	7-Hexadecene	+	+	++
11.0445	Hexadecane	-	+	+
11.0732	Cadina-1(10),6,8-triene	++	+	-
11.4789	α-Corocalene	+	-	-
11.6675	3-Methyldiadamantane	-	-	+
12.0961	8-Heptadecene	+	-	+
12.159	Heptadecane	-	+	+
12.3648	6-Heptadecanal	-	-	+
12.6334	2-Methyl-7-octadecene	-	-	+
13.1707	1-Octadecene	+	+	+++
13.2449	Octadecane	-	-	+
13.4392	5-Octadecene	-	-	+
14.1995	1-Nonadecene	+	+	+
14.2566	Nonadecane	+	-	+
14.5308	Hexadecanoic acid, methyl ester	-	+	+
14.8166	11-Hexadecenoic acid	-	+	-
14.8224	11-Tetradecyn-1-ol acetate	+	-	-
14.828	3-Methyl-4,6-hexadecadiene	-	-	+
14.9138	1,6-Tridecadiene	-	-	+
15.1825	5-Eicosene	+	+	++
15.2396	Eicosane	+	+	+
16.1255	1-Heneicosene	+	+	+
16.1712	Heneicosane	+	+	+
17.0228	1-Docosene	+	+	+
17.0685	Docosane	+	-	+
17.8915	9-Tricosene	+	-	+
17.9316	Tricosane	+	-	+
18.7203	1-Tetracosene	+	+	+
18.7946	Tetracosane	+	-	+
19.5204	1-Pentacosene	+	-	-
19.5547	Pentacosane	+	-	-
20.292	1-Hexacosene	+	-	+
20.3548	Hexacosane	+	-	+
21.0521	Heptacosane	+	-	-
21.7494	1-Octacosene	+	-	-
21.8008	1-Nonacosene	+	-	-

	22.4581	1-Triacontene	+	-	-
	23.1553	1-Hentriacontene	+	-	-
Nitrogen compound	4.0032	Benzonitrile	-	++	-
	5.6208	Benzyl nitrile	+	+	+
	6.8324	Benzenepropanenitrile	+	+	+
	14.3079	Tetradecanenitrile	-	+	+
Peptide	2.2887	Pyridine	+++	+++	+++
	2.5231	Pyridine-d5	+++	+++	-
	3.003	Pyridine, 4-methyl-	-	++	+++
	4.1291	1H-Indole	++	-	-
	4.5062	2-Cyanohex-3-ene	-	-	+
	4.5405	1H-Indene, 2,3-dihydro-	-	-	+
	4.632	1H-Indene	+	++	-
	5.8608	1H-Indene, 1-methyl-	+	++	++
	5.895	1H-Indene, 1-methylene-	-	+	+
	7.3982	1H-Inden-1-one, 2,3-dihydro-	-	-	+
	7.5125	Indole	+	-	-
	7.5181	Indolizine	-	+	+
	10.3015	1,1,4,5,6-Pentamethyl-2,3-dihydro-1H-indene	-	+	+
Polysaccharide	2.1515	2-Vinylfuran	-	++	-
	2.6145	2-Furancarboxaldehyde	++	++	+
	2.7517	Furfural	+++	+++	+++
	2.8431	1H-Pyrrole, 2-methyl-	++	-	+++
	2.9573	Furan-2-propyl	-	+	-
	3.0488	Cyclopent-2-en-1,4-dione	-	++	-
	3.1289	4-Cyclopenten-1,3-dione	++	++	-
	3.3632	2(5H)-furanone	++	+++	-
	3.426	2(3H)-Furanone, 5-methyl-	-	++	-
	3.7747	2-Furancarboxaldehyde, 5-methyl-	+++	+++	-
	4.1347	Benzofuran	-	+++	-
	5.3179	Benzofuran, 2-methyl-	+	+	+
	5.7808	Benzofuran, 2,3-dihydro-2-methyl	+	-	-
	6.4438	1,4:3,6-Dianhydro-*α*-*d*-glucopyranose	+	+	+
	6.5351	2,3-dihydro-benzofuran	+	+++	+
	12.4677	Psoralene	+	-	-

+ = Low relative abundance; ++ = medium relative abundance; +++ = high relative abundance; and - = null. *Retention time.

Table S2. List of compounds identified by analytical pyrolysis (Py-GC/MS) in the burned Amazon Forest (BAF), native Amazon Forest (NAF), and Brachiaria (BRA) soil samples at 5-10 cm soil depth.

Family	Rt* (min)	Name of compound	NAF	BRA	BAF
Aromatic	1.98	Benzene	+++	++	+++
	2.42	Benzene, methyl-	+++	+++	+++
	3.0373	Benzene, 1,2-dimethyl-	+++	-	-
	3.2145	Styrene	+++	++	+++
	3.7289	Benzene, propyl-	+	+	+
	3.7974	Benzaldehyde	+++	-	-
	3.906	Phenol	++	+++	++
	4.0889	Mesitylene	++	-	+++
	4.3804	Benzene, 1,3-diethyl-	++	-	-
	4.4032	Benzene, 1-ethenyl-3-methyl-	-	-	+++
	4.5404	Indane	+	+	-
	4.5861	Benzeneacetaldehyde	+	+	+
	4.6376	Indene	+	++	++
	4.7062	Benzene, butyl-	+	-	++
	4.832	Phenol, 4-methyl-	-	++	-
	4.8376	Ethanone, 1-phenyl-	-	-	++
	4.8433	Acetophenone	++	-	-
	4.9235	1,3-Benzenediol, 2-methyl	-	1.11	-
	4.9291	1H-Indene, 2,3-dihydro-1-methyl	+	-	-
	4.9291	1-Phenyl-1-butene	-	-	+
	5.0777	Mequinol	-	-	++
	5.6664	Benzene, 1-ethyl-4-(1-methylethyl)-	+	-	-
	5.7921	Benzene, (1-methyl-2-cyclopropen-1-yl)-	-	-	++
	5.9521	2-Phenylpentane	-	-	+
	6.038	Ethanone, 1-(2-methylphenyl)	-	+	-
	6.8437	Benzene, (1-ethylbutyl)	-	-	+
	7.0437	Benzene, hexyl-	+	+	+
	8.3125	Benzene, heptyl-	+	+	+
	8.8269	1H-Inden-1-one, 2,3-dihydro-3,4,7-trimethyl	+	-	-
	9.3529	Benzene, 1,5-cyclohexadien-1-yl	-	+	-
	9.5699	Benzene, octyl	+	+	+
	9.6327	Benzene, (1-cyclohexylethyl)	-	-	+
	10.1586	Benzene, 1,3,5-trimethyl-2-(3-methyl-1,3-butadienyl)	+	+	+
	10.793	Benzene, nonyl	+	+	+
	11.6389	Benzene, 1,2-dimethyl-4-(phenylmethyl)	+	-	-
	11.9591	Benzene, decyl	-	+	+
PAH	5.8951	Naphthalene	++	+++	+++
	5.9465	Naphthalene, 1,2,3,4-tetrahydro	-	+	-
	7.1352	Naphthalene, 1,2-dihydro-3-methyl-	-	-	+
	7.1925	Naphthalene, 2-methyl-	++	+++	++
	7.9524	Naphthalene, 1,2,3,4-tetrahydro-1,1-dimethyl	-	-	+
	8.5926	Biphenyl	+	-	-
	8.8212	Naphthalene, 1-(2-propenyl)	-	-	+
	8.8213	2,3-Dihydro-1H-Phenalene	-	+	-
	8.9298	Naphthalene, 1,2-dihydro-2,5,8-trimethyl-	+	-	-
	9.1012	Naphthalene, 2,6-dimethyl-	++	+	+
	9.3699	6N-Butyl-1,2,3,4-Tetrahydronaphthalene	+	-	-

	9.9871	Naphthalene, 1,2,3,4-tetrahydro-5-methyl-1-(1-methylethyl)-	-	-	+
	10.17	1,1,6,8-Tetramethyl-1,2-dihydro-naphthalene	-	-	+
	10.6444	Naphthalene, 1,2,3,4-tetrahydro-1-methyl-8-(1-methylethyl)	+	-	-
	11.0789	9H-Fluorene	-	++	+
	12.0961	Naphthalene, 1,6-dimethyl-4-(1-methylethyl)-	+++	+	-
	12.7647	1,4,5,8-Tetramethylnaphthalene	-	-	+
	12.7705	1,6-Dimethyl-3-ethylnaphthalene	+	-	-
	13.2964	9H-Fluorene, 9-methylene-	-	+	-
Lignin	5.0549	Phenol, 2-methoxy-	-	++	-
Lipid	3.1289	Protoanemonine	-	++	-
	3.9918	1-Decene	++	+++	+++
	4.2032	Cyclopropanecarboxylic acid, 3-ethenyl-2,2-dimethyl	-	-	+
	4.2834	Cycloheptanone	-	++	-
	5.0034	5-Undecene	+	++	++
	5.0891	Undecane	+	-	-
	5.152	9-Cycloheptadecen-1-one, (Z)-	-	-	+
	5.8035	1(7),5,8-*o*-Menthatriene	++	-	-
	6.1407	4-Dodecene	++	+	++
	7.2152	1-Tridecene	++	+	+
	7.4438	Tridecane	+	+	+
	8.5813	1-Tetradecene	-	++	++
	8.6726	Tetradecane	+	+	+
	8.7297	7-Tetradecene, (Z)-	-	-	+
	9.7928	1-Pentadecene	+	++	+
	9.8557	Pentadecane	+	+	+
	10.3358	trans-Calamenene	+++	+	-
	10.5129	α-Dehydro-ar-himachalene	+	-	-
	10.5872	α-Calacorene	+	+	-
	10.7073	Cyclopentene, 1,2-dimethyl-4-methylene-3-phenyl-	+	-	-
	10.9644	7-Hexadecene	+	+	+
	11.0444	Hexadecane	-	-	+
	11.073	Cadina-1(10),6,8-triene	++	-	-
	11.4788	α-Corocalene	+	-	-
	11.9589	1-Heptadecene	+	-	+
	12.1648	Heptadecane	-	+	+
	12.6276	1-Nonadecene	+	-	-
	12.6276	Cyclohexane, 1,2,3-trimethyl-	-	-	+
	13.1705	15-Heptadecenal	-	-	++
	13.1706	5-Octadecene	+	+	+
	13.2336	Octadecane	-	+	+
	14.1937	1-Nonadecene	-	+	+
	14.2566	Nonadecane	-	+	+
	14.308	2-Heptadecanone	-	+	-
	14.5309	Hexadecanoic acid, methyl ester	-	+	-
	14.8167	Oxacycloheptadecan-2-one	-	+	-
	15.2396	5-Eicosene	+	+	++
	15.2852	Eicosane	-	+	+
	16.1255	1-Heneicosene	-	+	+
	16.1712	Heneicosane	-	+	+
	17.0228	1-Docosene	-	+	+
	17.0685	Docosane	-	+	+
	17.8914	9-Tricosene	-	-	+

	17.9314	Tricosane	-	-	+
	18.7203	1-Tetracosene	-	+	+
	18.7946	Tetracosane	-	+	+
	19.5545	Pentacosane	-	-	+
	20.2918	9-Hexacosene	-	-	+
	20.3604	Hexacosane	-	-	+
	21.0634	Heptacosene	-	-	+
	21.7606	1-Octacosene	-	-	+
	21.8064	Octacosane	-	-	+
Nitrogen compound	2.1743	Acetamide, *N*-2-propynyl-	++	-	-
	4.026	Benzonitrile	+++	-	-
	4.2146	3-Pyridinecarbonitrile	++	-	-
	5.375	*o*-Methoxybenzonitrile	-	+	-
	5.6378	Benzyl nitrile	+	+	+
	6.8494	Benzenepropanenitrile	+	+	-
Peptide	2.5116	Pyridine-d5	-	+++	+++
	2.9974	Pyridine, 3-methyl	-	++	+++
	3.5402	*N*-Methylmaleimide	+	-	-
	3.6661	Pyridine	+++	+++	+++
	3.666	Pyridine, 2,5-dimethyl-	-	-	+
	4.4148	Benzanamine, *N*-methyl	-	++	-
	5.3749	2-Methyl-6-*cis*-propenylpyrazine	-	-	+
	5.6663	1H-Indene, 2,3-dihydro-5-methyl-	-	-	+
	5.7922	2-Methylindene	-	++	-
	5.9521	1-Amino-2-hydroxyindane	+	-	-
	6.5524	1H-Pyrrolo[2,3-b]pyridine, 3,4-dimethyl	-	+++	-
	7.2554	5-Methylquinoxaline	-	+	-
	7.3868	1H-Inden-1-one, 2,3-dihydro-	-	+	+
	7.5297	Indolizine	-	+	+
	10.3015	1H-Indene, 2,3-dihydro-1,1,2,3,3-pentamethyl-	-	-	+
	10.3016	1,1,3,3,5-Pentamethyl-2,3-dihydroindene	-	+	-
	12.4963	Pyrazolo[5,1-c][1,2,4]benzotriazin-8-ol	-	+	+
Polysaccharide	2.1402	2-Vinylfuran	-	++	-
	2.2657	1H-Pyrrole, 1-methyl-	++	-	-
	2.6315	Furfural	+++	+++	+++
	2.8317	1H-Pyrrole, 2-ethyl-	-	+++	+++
	3.1459	4-Cyclopentene-1,3-dione	++	++	-
	3.3289	2-Acetylfuran	-	+++	++
	3.3516	2-Cyclopenten-1-one, 3,4-dimethyl	+++	-	-
	3.7747	2-Furancarboxaldehyde, 5-methyl-	-	+++	+++
	4.1291	Benzofuran	-	+++	-
	4.1919	1H-Pyrrole-2-carboxaldehyde	-	+	-
	4.4891	1H-Pyrrole, 2,5-dimethyl-	-	+	-
	5.255	1H-Pyrrolo[2,3-b]pyridine, 1-methyl	-	+	+

+ = Low relative abundance; ++ = medium relative abundance; +++ = high relative abundance; and - = null. *Retention time.

Table S3. List of compounds identified by analytical pyrolysis (Py-GC/MS) in the burned Amazon Forest (BAF), native Amazon Forest (NAF), and Brachiaria (BRA) soil samples at 40-50 cm soil depth.

Family	Rt* (min)	Name of compound	NAF	BRA	BAF
Aromatic	1.9401	Benzene	+++	+++	+++
	2.3916	Toluene	+++	-	+++
	2.5345	Ethanone. 1-cyclopentyl	-	-	+++
	2.9803	Benzene, 1,3-dimethyl-	+	-	+
	3.0375	*p*-Xylene	+	+	+
	3.2146	Styrene	++	++	-
	4.0948	Benzene, 1,2,3-trimethyl-	+	++	-
	4.649	Benzene, (1-methylpropyl)	-	+	-
PAH	6.3121	Naphthalene	-	+	-
Lipid	3.5345	4-Decene	-	++	-
	3.9517	4-Undecene, (E)-	-	+	-
	4.9347	1-Dodecene	-	+	-
	2.0544	Cyclohexene, 4-methyl	+++	++	-
	2.9801	3-Heptene, 2,6-dimethyl-	-	+	-
	3.0773	Dimethyl-2,3-heptene-3	-	+	-
	3.7974	4-Octene, 2,3,6-trimethyl	-	+	-
	4.3863	1,3-Cyclopentadiene, 5-(1-methylpropylidene)	+	-	-
Peptide	1.8829	2H-Pyran-5,6-dihydro-2-methyl	++	-	-
	2.1573	1-Vinylimidazole	++	-	-
	2.2544	1H-Pyrrole, 1-methyl-	++	+++	++
	2.3171	1H-Pyrrole	-	+++	-
	2.3287	Pyridine	+++	-	-
	2.8887	2-Buten-1-amine, N-butyl	-	++	-
Polysaccharide	1.7515	Furan, 2-methyl-	+++	-	-
	2.0658	Furan, 2-ethyl	-	-	+++

* + = Low relative abundance; ++ = medium relative abundance; +++ = high relative abundance; and - = null. *Retention time.
